# Mitophagy in pathogenesis and therapeutic implications for infection

**DOI:** 10.1038/s12276-026-01852-7

**Published:** 2026-10-05

**Authors:** Bokeum Jung, Bomi Lee, Asmita Sapkota, Taylor Roh, Wonhyoung Seo, Jin Kyung Kim, Eun-Kyeong Jo

**Affiliations:** 1 Department of Medicine, College of Medicine, Chungnam National University, Daejeon, Republic of Korea; 2 Department of Microbiology, College of Medicine, Chungnam National University, Daejeon, Republic of Korea; 3 Department of Medical Science, College of Medicine, Chungnam National University, Daejeon, Republic of Korea; 4 Brain Korea 21 FOUR Project for Medical Science, Chungnam National University School of Medicine, Daejeon, Republic of Korea; 5 Department of Internal Medicine, Division of Hematology/Oncology, Chungnam National University Hospital, Daejeon, Republic of Korea; 6 Department of Microbiology, School of Medicine, Keimyung University, Daegu, Republic of Korea; 7 Biomedical Research Institute, Chungnam National University Hospital, Daejeon, Republic of Korea

**Keywords:** Infection, Innate immunity

## Abstract

Mitophagy, a critical selective autophagy pathway targeting dysfunctional mitochondria, is involved in cellular homeostasis and quality control under various stressors. During infection, invading pathogens cause mitochondrial abnormalities that trigger mitophagy. Most bacteria and viruses can exploit mitophagy as a pro-pathogenic process to escape host antiviral responses during infection. Emerging evidence suggests that restoring mitophagy is beneficial for mitigating excessive inflammation, such as in sepsis, whereas mitophagy blockade can activate host defense during bacterial and viral infections. Here we review current knowledge on the role of mitophagy in bacterial and viral infections and sepsis, focusing on the mechanisms by which pathogens evade mitophagy or influence pathogenesis through mitophagy during different types of infection. We also highlight the potential beneficial effects of mitophagy modulators, reagents and small molecules against sepsis-induced pathologies and infections. Progress in these areas will expand our knowledge on alleviating pathology, promoting antimicrobial immune responses and maintaining cellular homeostasis, ultimately providing potential preventive and therapeutic strategies against pathogenic infections and inflammation associated with dysregulated mitophagy.

## Mitophagy’s role in infection and inflammation explored

Mitophagy, a selective autophagy process, is crucial for regulating mitochondrial homeostasis and shaping infection pathogenesis. This Review explores the dual role of mitophagy in bacterial and viral infections and how pathogens exploit it for survival. The authors detail mechanisms of canonical and noncanonical mitophagy pathways, emphasizing the molecular mechanisms underlying PINK1/Parkin-dependent and alternative mitophagy. They also discuss how pathogens such as *Staphylococcus aureus* and *Helicobacter pylori* manipulate mitophagy to evade immune responses, while viruses such as SARS-CoV-2 and influenza A virus use it to suppress antiviral defenses. The Review underscores the therapeutic potential of targeting mitophagy, suggesting that modulating this process could lead to novel treatments for infections and sepsis. Future research should focus on understanding pathogen-specific interactions with distinct mitophagy pathways to develop targeted therapies.

This summary was initially drafted using artificial intelligence, then revised and fact-checked by the author.

## Introduction

Autophagy, a well-known catabolic process, degrades damaged or dysfunctional proteins and macromolecules via lysosomes to maintain cellular homeostasis^1–3^. Autophagy was previously considered a nonselective bulk degradation process. However, accumulating evidence shows that activation of selective autophagy enables cells to recognize and degrade specific targets, including damaged organelles, aggregated proteins and intracellular bacteria. Recent research has elucidated distinct activation mechanisms of selective autophagy pathways, including mitophagy, endoplasmic reticulum (ER)-phagy, nucleophagy and xenophagy. Among them, mitophagy via the lysosomal degradation of mitochondria is crucial for preserving cellular function and integrity under stress^4–6^.

Human mitochondria are organelles that possess their own highly compact genomes that lack introns^7^. In addition to the principal bioenergetic function of ATP synthesis, mitochondria play crucial roles in maintaining various cellular processes, including redox equilibrium, iron metabolism, biosynthesis of principal metabolites, regulation of cellular calcium homeostasis, immune functions and programmed cell death^8–10^. In the context of pathogenic infections, mitochondria also help to regulate antimicrobial responses, energy metabolism, inflammation and apoptosis, thereby impacting host defense and infection outcomes^11,12^. Dysregulated mitophagy results in the accumulation of dysfunctional mitochondria, leading to severe infection and other human pathologies, including neurodegeneration, cancer, aging and autoimmune diseases^13–17^. In addition, the release of mitochondrial DNA (mtDNA) into the cytoplasm, which is associated with defective mitophagy or autophagy, triggers specific inflammasome activation by inducing innate immune nucleic acid receptors and activating downstream signaling cascades^11,18^. The aberrant activation of mtDNA-driven immune responses involving several DNA-sensing receptors is associated with the pathogenesis of viral and bacterial infections^11^.

During infection, mitochondria undergo metabolic reprogramming, whereby changes in mitochondrial dynamics, such as fission, fusion, ionic fluxes, and mtDNA synthesis and release, activate mitophagy to maintain mitochondrial homeostasis. However, numerous pathogens can co-opt mitophagy to favor their pathogenesis and intracellular replication, as well as evade host immune responses during infection. In this Review, we discuss the role of mitophagy in bacterial and viral infections and sepsis. First, we summarize the mitophagy process and provide an overview of how mtDNA release activates specific innate immune pathways via DNA-sensing receptors. We then summarize recent findings on pathogen-induced interactions with host mitophagy machinery and the role of mitophagy in pathogenesis during three distinct types of infection. Finally, we highlight the therapeutic potential of mitophagy modulation for preventing and ameliorating pathogen-induced infections, sepsis and sepsis-related pathological conditions. This research broadens our understanding of the crosstalk between mitophagy and immune defense systems and suggests new avenues for developing therapeutic strategies against infectious and inflammatory diseases.

## Overview of mitophagy

**Fig. 1:**
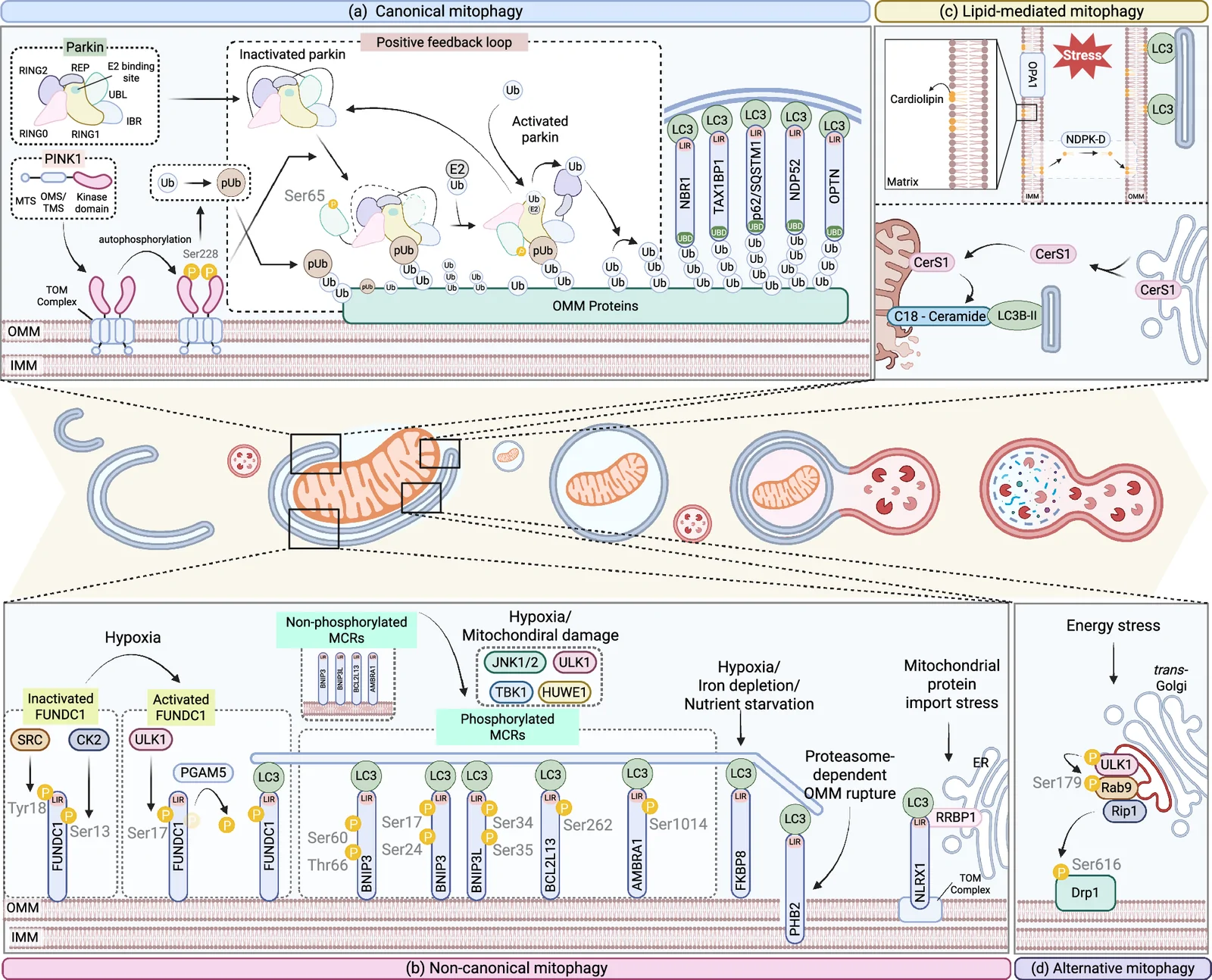
Mechanisms of mitophagy. **a**, Canonical mitophagy: mitochondrial depolarization stabilizes PINK1 on the OMM, where it recruits and activates the E3 ubiquitin ligase Parkin. Parkin-mediated ubiquitination of OMM proteins promotes the recruitment of selective autophagy receptors, including NBR1, TAXBP1, p62/SQSTM1, NDP52 and OPTN. These receptors contain LIRs and ubiquitin-binding domains (UBDs), enabling them to bridge ubiquitinated mitochondria with LC3-positive autophagosomal membranes. **b**, Noncanonical mitophagy: receptor-mediated mitophagy occurs independently of ubiquitination. Mitochondrial proteins, including FUNDC1, BNIP3, BNIP3L, BCL2L13, Autophagy and Beclin 1 Regulator 1 (AMBRA1), FK506-binding protein 8 (FKBP8), PHB2 and NLRX1, function as mitophagy receptors through their LIR motifs, thereby directly linking damaged mitochondria to the autophagic machinery. **c**, Lipid-mediated mitophagy: specific mitochondrial lipids act as mitophagy signals. CL is translocated from the inner mitochondrial membrane (IMM) to the OMM through the action of NDPK-D, whereas CerS1 generates C18-ceramide at the mitochondrial surface. Both CL and ceramide directly interact with LC3 to facilitate mitophagy. **d**, Alternative mitophagy: under conditions of energy stress, an LC3-independent mitophagy pathway is activated. This pathway utilizes trans-Golgi network-derived vesicles and is mediated by the ULK1–Rab9–RIP1 signaling complex. Created in BioRender. Lee, B. (2026) https://BioRender.com/8bl9em0.

**Fig. 2:**
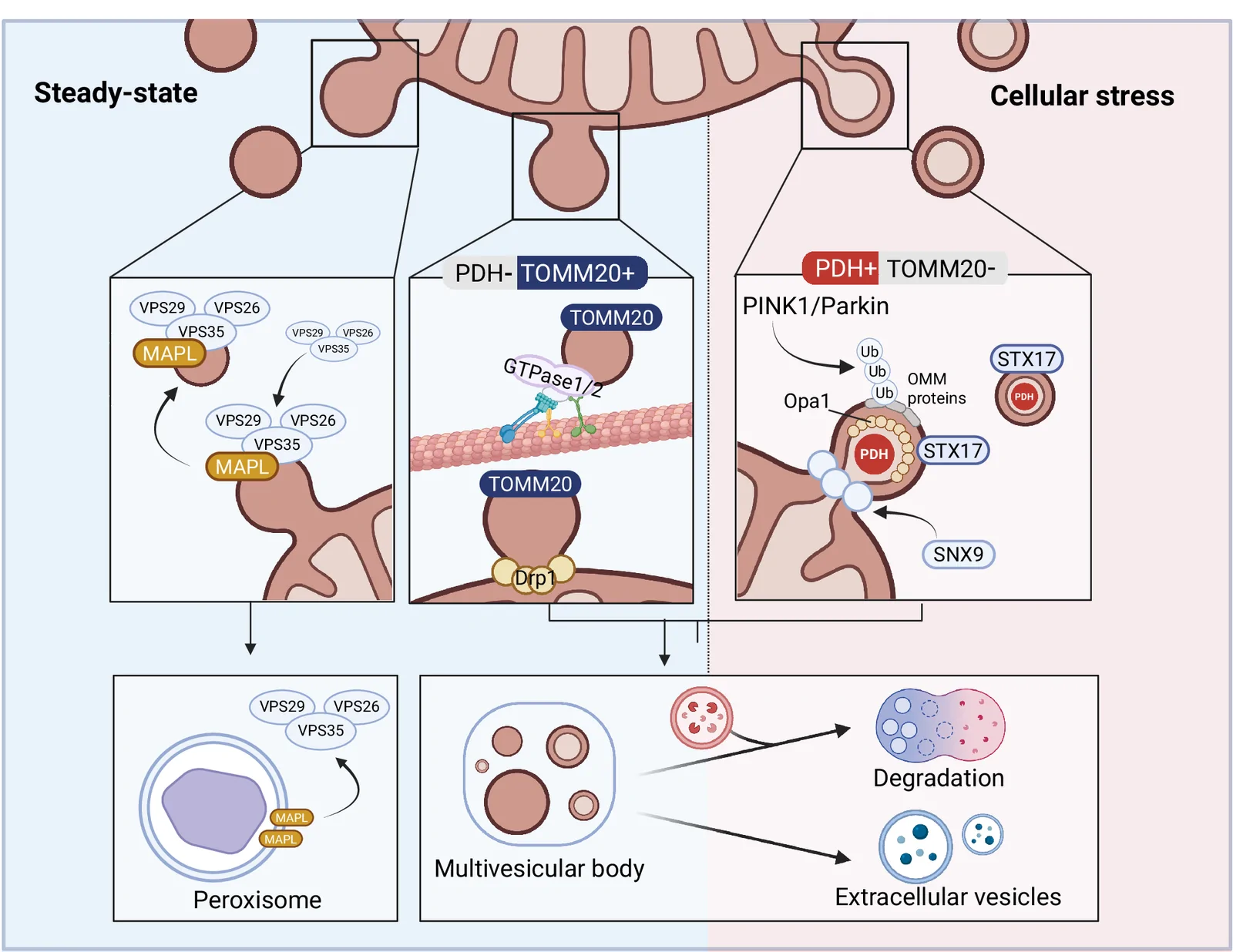
Biogenesis and trafficking mechanisms of MDVs. Under basal conditions, mitochondrial-anchored protein ligase-positive MDVs are transported to peroxisomes through the VPS complex (VPS35, VPS29 and VPS26). Meanwhile, translocase of outer mitochondrial membrane 20 (TOMM20)-positive and pyruvate dehydrogenase (PDH)-negative (TOMM20⁺PDH⁻) MDVs are generated in a DRP1-dependent manner and delivered to multivesicular bodies along microtubules via GTPase1/2. In response to cellular stress, PINK1/Parkin-mediated ubiquitination of OMM proteins promotes the formation of PDH-positive and TOMM20-negative (PDH⁺TOMM20⁻) MDVs through a process involving optic atrophy 1(OPA1), syntaxin 17 and sorting nexin 9. These stress-induced MDVs are subsequently trafficked to multivesicular bodies, where they are either targeted for lysosomal degradation or released as extracellular vesicles. Created in BioRender. Lee, B. (2026) https://BioRender.com/8bl9em0.

**Fig. 3:**
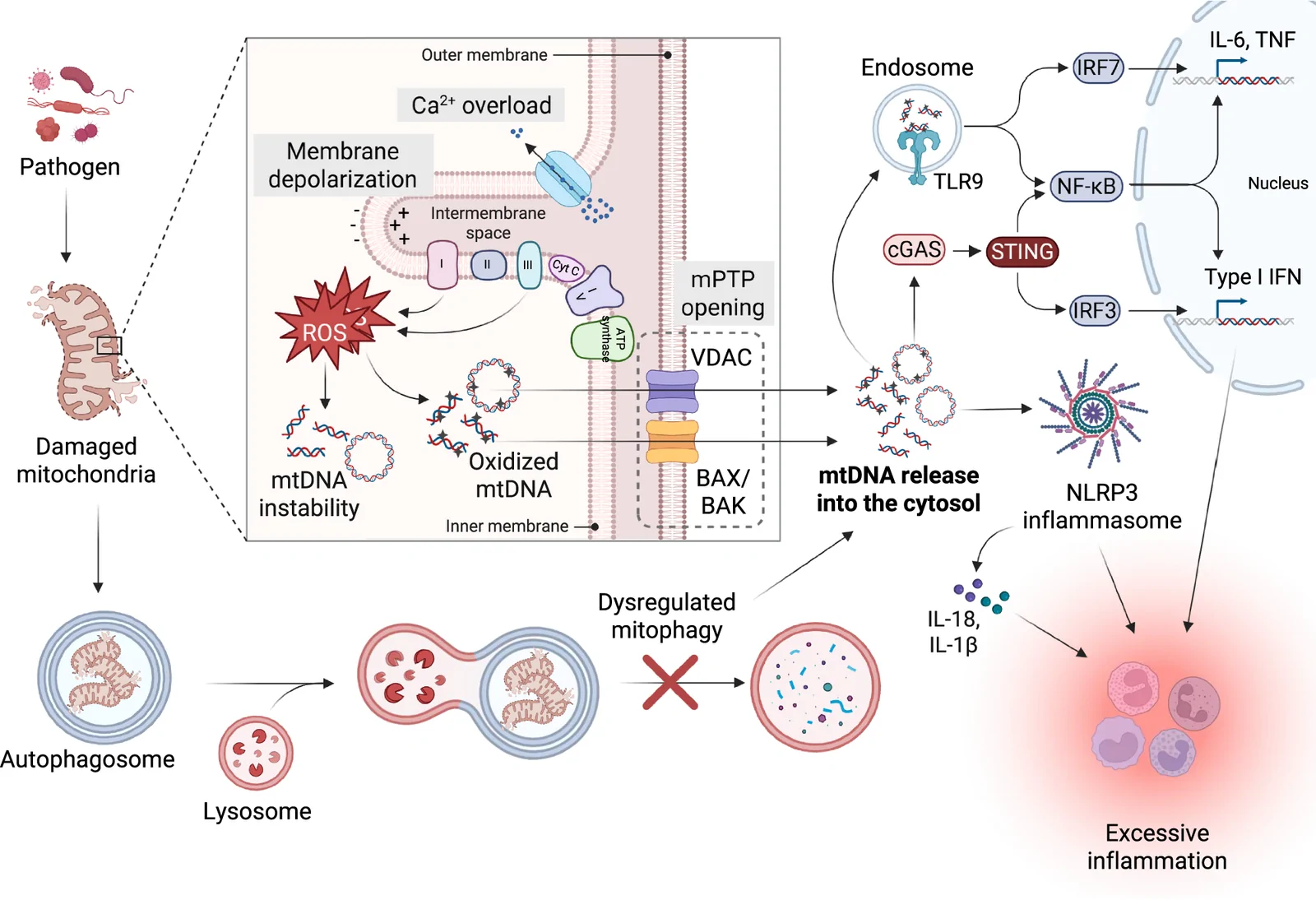
mtDNA-derived signals drive excessive inflammatory responses. During infection, impaired mitophagy, excessive mtROS production, mitochondrial Ca²⁺ overload, mitochondrial membrane depolarization, and the opening of the mitochondrial permeability transition pore (mPTP) through VDAC and BAX/BAK promote the release of destabilized or oxidized mtDNA into the cytosol. Cytosolic mtDNA activates the cGAS–STING pathway and is recognized by Toll-like receptor 9 within endosomes, thereby initiating downstream inflammatory signaling. In addition, mtDNA serves as a potent activator of the NLRP3 inflammasome. Collectively, these mtDNA-driven signaling pathways amplify inflammatory responses and contribute to pathological inflammation. Created in BioRender. Kim, J. K. (2026) https://BioRender.com/b89ms7z.

Mitophagy, a selective form of autophagy responsible for clearing damaged mitochondria, is essential for maintaining mitochondrial quality during infection and inflammation^14,19^. Mechanistically, mitophagy can be triggered through multiple pathways, currently classified into canonical and noncanonical pathways^20^. The canonical pathway predominantly involves the well-studied phosphatase and tensin homolog (PTEN)-induced kinase 1 (PINK1)/Parkin pathway, wherein Parkin-mediated ubiquitination plays a central role in the clearance of depolarized mitochondria^20^. Conversely, receptor-mediated noncanonical mitophagy depends on specific receptor proteins located directly on the outer mitochondrial membrane (OMM)^21^. In this section, we provide a brief overview of canonical and noncanonical mitophagy pathways, which encompass proteinaceous mitochondrial cargo receptor (MCR)-dependent and lipid receptor-mediated pathways. Furthermore, we discuss the formation of mitochondria-derived vesicles (MDVs), an initial mitochondria quality control mechanism that is activated before mitophagy^22^ (Figs. 1 and 2). We also summarize the impact of mtDNA release due to defective mitophagy in the context of infection (Fig. 3).

### Canonical and noncanonical mitophagy pathways

#### Canonical mitophagy pathways

The canonical PINK1/Parkin pathway involves PINK1 translocation and stabilization in the mitochondrial OMM, which facilitates Parkin activation. In this process, PINK1—a serine/threonine ubiquitin kinase—phosphorylates Parkin on S65 to augment the recruitment of active Parkin to the OMM^23–27^. Activated Parkin then ubiquitinates numerous proteins residing on the OMM, such as mitofusin-2 and voltage-dependent anion channel-1 (VDAC1). The resulting ubiquitin chains are recognized by specialized cargo receptors, including p62 (also known as sequestosome 1, SQSTM1), neighbor of BRCA1 gene 1, nuclear dot protein 52 kDa (NDP52), Tax-1 binding protein 1, and optineurin (OPTN), all of which harbor both a ubiquitin-binding domain and a C-terminal microtubule-associated protein 1 light chain 3 (LC3)-interacting region (LIR), ultimately driving the selective autophagic removal of damaged mitochondria^16,28–30^ (Fig. 1a).

#### Noncanonical mitophagy pathways

Noncanonical receptor-mediated mitophagy is regulated by several MCRs, including FUN14 domain-containing 1 (FUNDC1), BCL2-interacting protein 3 (BNIP3), BCL2-interacting protein 3-like (BNIP3L, also known as NIX or NIP3-like protein X), BCL2L13, Autophagy and Beclin 1 Regulator 1, FK506-binding protein 8, prohibitin 2 (PHB2) and NLR family member X1 (NLRX1)^31^. These receptors promote mitophagy by directly interacting with LC3 and gamma-aminobutyric acid receptor-associated protein (GABARAP) family proteins through their LIR motifs in response to mitochondrial stress^31^. Among them, FUNDC1 is one of the best-characterized mitophagy receptors. Under basal conditions, FUNDC1-mediated mitophagy is suppressed by casein kinase 2 and Src kinase through phosphorylation of Ser13 and Tyr18, respectively. Upon mitochondrial stress, such as hypoxia or carbonyl cyanide *p*-(trifluoromethoxy) phenylhydrazone treatment, phosphoglycerate mutase family member 5 (PGAM5) dephosphorylates FUNDC1 at Ser13, while unc-51 like autophagy activating kinase 1 (ULK1) phosphorylates Ser17^32,33^. Thus, the orchestrated post-translational modifications of FUNDC1 by PGAM5, ULK1 and other kinases act as a crucial regulatory switch that senses mitochondrial damage and activates mitophagy^32^.

In response to hypoxia and/or mitochondrial damage, BNIP3 and BNIP3L—members of the BH3-only protein family—function as mitophagy receptors by directly interacting with LC3 and GABARAP family proteins^34,35^. Their mitophagic activity depends on phosphorylation of BNIP3 at Ser17/24 and BNIP3L at Ser34/35, with ULK1 serving as a key upstream kinase^35,36^. Another MCR, BCL2L13, promotes mitochondrial fission and mitophagy. Although initially reported to function independently of Dynamin-related protein 1 (DRP1)^37^, recent studies have shown that BCL2L13 enhances DRP1 phosphorylation at Ser616, thereby stimulating mitochondrial fragmentation and mitophagy^38^. Additional noncanonical mitophagy regulator Autophagy and Beclin 1 Regulator 1 interacts with LC3 through its conserved LIR motif and promotes mitophagy in both PARKIN-dependent and PARKIN-independent settings^39^. FK506-binding protein 8 recruits LC3A to damaged mitochondria and facilitates PARKIN-independent mitophagy while escaping autophagic degradation through translocation to the ER^40^. Upon mitochondrial outer membrane permeabilization or mitophagy-induced stress, the OMM is disrupted in a Parkin- and proteasome-dependent manner, exposing PHB2 to the cytosol. PHB2 then directly interacts with LC3 via its LIR motif, promoting mitophagy^41^. Furthermore, the mitochondrial protein NLRX1 functions as a mitophagy receptor by interacting with LC3 and the ER-resident ribosome-binding protein 1, thereby facilitating LC3 lipidation and autophagosome formation at ER-mitochondria contact sites (Fig. 1b).

Recent studies have identified mitochondrial lipids as noncanonical mitophagy receptors. Upon mitochondrial damage, cardiolipin (CL)—a phospholipid typically confined to the IMM—is externalized to the OMM by NDPK-D (also called NM23-H4 and encoded by the *NME4* gene) for mitophagy initiation^42^. NDPK-D-mediated CL externalization may also be regulated through its interaction with the dynamin-like GTPase Optic Atrophy 1, linking mitochondrial dynamics to mitophagy^42^. In addition to CL, ceramides mediate mitophagy in response to specific stress. C18-ceramides preferentially interact with the C18-ceramide-binding region of LC3-II, whereas microtubule-associated protein 1 light chain 3 beta (LC3B)-II can also bind C16- and C24-ceramides in response to CerS1 activation, thereby facilitating mitochondrial clearance^43^. Key residues within LC3B, including Ile35 and Phe52, are required for ceramide binding^43^ (Fig. 1c). Distinct from these LC3-dependent pathways, alternative mitophagy proceeds through ULK1/Rab9-mediated trafficking of trans-Golgi network-derived vesicles. This pathway bypasses LC3 lipidation and selectively targets damaged mitochondria for degradation^44,45^ (Fig. 1d).

#### MDVs

MDVs are specialized carriers that transport mitochondrial proteins and lipids to lysosomes, peroxisomes or multivesicular bodies and can also be released as extracellular vesicles^46,47^. Their biogenesis is regulated through cargo-selective pathways. Under cellular stress, double-membrane MDVs containing inner membrane and matrix components are generated through a PINK1/Parkin-dependent pathway involving optic atrophy 1, sorting nexin 9 and syntaxin 17^47–49^. By contrast, under basal conditions, single-membrane MDVs enriched in the translocase of outer mitochondrial membrane 20 are formed through DRP1 and mitochondrial Rho GTPases 1 and 2^50^. Another distinct MDV population transports mitochondria-anchored protein ligase to peroxisomes via the vacuolar protein sorting (VPS)35 and VPS26^51,52^. Collectively, MDVs function as a first-line mitochondrial quality-control mechanism, termed micromitophagy, by selectively removing damaged mitochondrial compartments^51,52^. Dysregulated MDV trafficking has also been implicated in human diseases, including cancer metastasis^46,51^.

Although individual mitophagy pathways and their regulation are increasingly well characterized, how different mitochondrial stress signals, including pathogenic invasion, selectively activate specific pathways or coordinate multiple MCRs remains unclear. Moreover, the extent of functional overlap and cooperation among these mitophagy pathways has yet to be fully evaluated.

### mtDNA signals augment excessive inflammation during infections

Mitophagy is essential for maintaining immune homeostasis and preventing excessive inflammatory responses^17^. Various forms of mitochondrial stress, including defective mitophagy, excessive reactive oxygen species (ROS) production, mitochondrial Ca^2+^ overload, membrane depolarization and mitochondrial permeability transition pore opening, can promote the release of mtDNA into the cytosol^11,18^. Pathogen invasion frequently disrupts mitochondrial homeostasis, leading to mitochondrial damage, impaired organelle dynamics and accumulation of cytosolic mtDNA^53,54^.

Because mtDNA contains abundant hypomethylated cytidine–phosphate–guanosine motifs, its mislocalization acts as a potent danger signal that activates hyperinflammatory responses^11^. Cytosolic mtDNA is sensed by cyclic GMP–AMP synthase (cGAS), absent in melanoma 2, and Z-DNA binding protein 1, whereas endosomal mtDNA is recognized by Toll-like receptor 9^11,18^. In addition, both mtDNA and oxidized mtDNA activate NOD-like receptor protein 3 (NLRP3) inflammasomes^11^. Among these pathways, the cGAS–stimulator of interferon genes (STING) signaling induces interferon (IFN) regulatory factor 3 activation and robust type I IFN production while simultaneously promoting nuclear factor kappa B (NF-κB), signal transducer and activator of transcription (STAT) 6 and mitogen-activated protein kinase signaling, thereby amplifying inflammatory responses^11^. Consequently, excessive mtDNA-driven inflammation contributes to the pathogenesis of diverse infectious diseases caused by bacteria, viruses and protozoa^55^.

Emerging evidence indicates a close link between mitophagy dysfunction and pathological IFN responses. *PINK1-* or *PARK2-*deficient cells exhibit defective mitophagy, accumulation of cytosolic mtDNA, and exaggerated cGAS–STING-dependent type I IFN responses^56^. Similarly, impaired BNIP3L-dependent mitophagy promotes mitochondrial antiviral signaling protein (MAVS) aggregation and enhances the production of type I IFNs and inflammatory cytokines^57^. Although type I IFN responses are critical for antiviral immunity, excessive activation is associated with pathological inflammation and disease progression in active pulmonary tuberculosis^58–60^. Thus, defective mitophagy represents a key driver of mtDNA release, aberrant IFN signaling and inflammatory pathology during infection (Fig. 3). Future studies should clarify how mitophagy intersects with IFN signaling, NF-κB activation and inflammasome pathways across different infectious diseases and whether targeting mitochondrial quality-control pathways can be exploited for host-directed therapeutic interventions.

## Mitophagy in bacterial infection

**Fig. 4:**
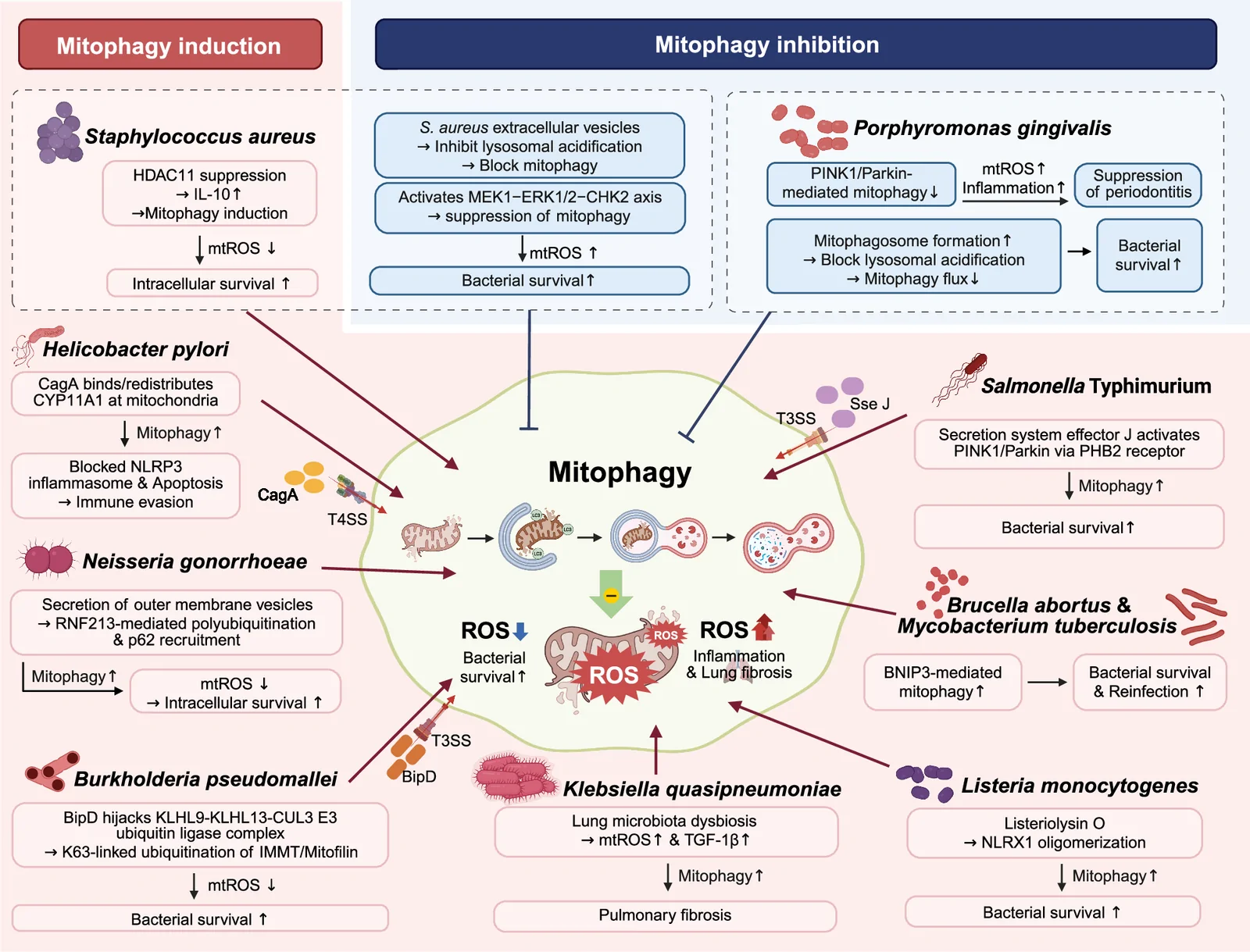
Roles of mitophagy in bacterial infections. During bacterial infection, pathogens manipulate host mitophagy to regulate mtROS production and shape infection outcomes. Numerous intracellular pathogens, including *Staphylococcus aureus*, *Helicobacter pylori*, *Burkholderia pseudomallei*, *Neisseria gonorrhoeae*, *Listeria monocytogenes*, *Salmonella enterica* serovar Typhimurium and *Mycobacterium tuberculosis*, actively induce mitophagy to facilitate the clearance of damaged mitochondria, thereby limiting mtROS accumulation and excessive inflammasome activation to promote intracellular survival. *Brucella abortus* exploits host mitophagy through a distinct mechanism that facilitates intracellular escape and cell-to-cell transmission. In contrast, other pathogens evade host defense by suppressing mitophagic flux. *Staphylococcus aureus* and *Porphyromonas gingivalis* inhibit mitophagy and lysosomal acidification, thereby preventing autophagic clearance and establishing persistent intracellular infection. Furthermore, during *Klebsiella quasipneumoniae* infection, mitophagy is triggered alongside mtROS production, driving TGF-β1-mediated pathological inflammation and progressive tissue fibrosis. Collectively, modulation of host mitophagy represents a central strategy by which bacterial pathogens promote survival, persistence, and disease progression. Created in BioRender. Lee, B. (2026) https://BioRender.com/y2n569z.

Numerous bacterial pathogens can exploit or manipulate mitophagy to evade host immune defense and drive infectious pathogenesis (Fig. 4). Existing research has not fully clarified whether mitophagy is a defensive host response or whether pathogens trigger host mitophagy to escape antibacterial responses^61^.

*Staphylococcus aureus* can activate mitophagy and scavenge mitochondrial ROS (mtROS) through the histone deacetylase (HDAC) 11 suppression-induced enhancement of interleukin (IL)-10, thereby promoting the intracellular survival of *S. aureus* within macrophages. These data suggest that the HDAC11–IL-10 axis promotes mitophagy by suppressing the mammalian target of rapamycin pathway during *S. aureus* infection^62^. In addition, *S. aureus* extracellular vesicles can disrupt mitochondria and induce apoptosis in bovine mammary epithelial cells; although mitophagy is activated in response, the clearance of damaged mitochondria is impeded due to the inhibition of the lysosomal acidic environment^63^. Moreover, in macrophages, *S. aureus* hyperactivates the mitogen-activated protein kinase 1 (MEK1)–ERK1/2 axis to suppress CHEK2 expression, leading to excessive mitophagy and decreased mtROS levels, which impairs bactericidal activity^64^.

In the case of *Helicobacter pylori* infection, the cytotoxin-associated gene A (CagA) protein of the bacterium redistributes the mitochondrial protein cytochrome P450 family 11 subfamily A member 1 and then binds with it, inhibiting mitophagy by accelerating mitochondrial cholesterol accumulation^65^—which is an effective strategy for evading the host immune system by blocking NLRP3 inflammasome activation and apoptosis of the infected cells^66^.

*Burkholderia pseudomallei*, a facultative intracellular bacterium, induces mitophagy through the type III secretion system needle tip protein, burkholderia invasion protein D (BipD). *B. pseudomallei* induces mitophagy by hijacking the Kelch-like 9–kelch-like 13–cullin3 E3 ubiquitin ligase complex, thus leading to K63-linked ubiquitination of the inner mitochondrial membrane protein (IMMT, also known as mitofilin)^67^. BipD-induced and p62-mediated mitophagy inhibit mtROS generation and promote the intracellular survival of *B. pseudomallei*^67^. Similarly, *Neisseria gonorrhoeae*, the bacterial pathogen inducing gonorrhea, secretes outer membrane vesicles that deliver the porin protein PorB to mitochondria. PorB induces mitophagy through dual mechanisms: it dissipates the mitochondrial membrane potential to activate the PINK1- and OPTN/NDP52-dependent pathway, and recruits the E3 ubiquitin ligase ring finger protein 213 (RNF213), leading to its K63-linked polyubiquitination, which induces mitophagy in a p62-dependent manner, thereby reducing mtROS production and favoring intracellular survival^68^.

During infection with *Salmonella enterica* serovar Typhimurium, a major zoonotic pathogen of public health concern, the bacterial type III secretion system effector SseJ activates the PINK1/Parkin-mediated mitophagy pathway via the mitophagy receptor PHB2. Effector SseJ-induced mitophagy promotes bacterial survival and persistence, whereas inhibition of mitophagy attenuates the pathogenicity of *S*. Typhimurium^69^. Moreover, modulation of phospho-TANK-binding kinase 1-mediated mitophagy restricts intracellular *S*. Typhimurium proliferation^70^. A recent study showed that bleomycin exposure induces lung microbiota dysbiosis with an overgrowth of tissue-resident *Klebsiella quasipneumoniae*, accompanied by elevated ROS, macrophage mitophagy and increased TGF-β1—crucial factors inducing fibrosis progression. These findings suggest that mitophagy activation is critically linked to lung microbiota dysregulation and the development of pulmonary fibrosis^71^.

*Listeria monocytogenes* is one of the bacterial species that causes listeriosis^72^. The virulence factor listeriolysin O, a key molecule in the pathogenicity of *L. monocytogenes*, triggers oligomerization of the NLRX1—which in turn induces host mitophagy^73^. Listeriolysin O-mediated mitophagy in *L. monocytogenes* promotes bacterial survival and prevents bacterial death^73^.

In *Mycobacterium tuberculosis* infection, Atg5 in CD11c^+^ cells is not directly involved in bacterial growth control but in the control of proinflammatory cytokine generation and neutrophil infiltration, depending on autophagy rather than mitophagy^74^. Conversely, specific mitophagy involving BNIP3 has been linked to *M. tuberculosis* infection. In macrophages, BNIP3 is required for mitophagy activation, which promotes intracellular *M. tuberculosis* survival by decreasing mtROS production^75^. However, the exact roles of BNIP3 and other mitophagy genes in virulent strains of *M. tuberculosis* remain unclear. Meanwhile, during infection with *Brucella abortus*, a facultative intracellular pathogen, BNIP3L-mediated mitophagy is required for bacterial egress, and BNIP3L silencing reduces bacterial burden during reinfection^76^.

However, during infection with *Porphyromonas gingivalis*, mitophagy functions in the opposite manner. *P. gingivalis* is a major causative agent of periodontitis and has been associated with several systemic diseases^77^. PINK1/Parkin-mediated mitophagy attenuates *P. gingivalis*-induced inflammation by limiting mitochondrial damage and mtROS production^78^. The anti-inflammatory effects of mitophagy inhibit the progression of periodontitis, suggesting mitophagy as a potential therapeutic target^78^. However, a recent study has suggested that *P. gingivalis* regulates mitophagy and lysosomal function by disrupting autophagy/mitophagy flux. In direct infections targeting endothelial cells, progressive mitophagy with missing lysosomal function aids *P. gingivalis* intracellular survival by blocking xenophagosome formation and autophagosome/xenophagosome–lysosome fusion^79^. A schematic overview of how various bacterial pathogens modulate distinct mitophagy pathways is shown in Fig. 4, whereas detailed information is provided in Table 1.

**Table 1:** Mitophagy in bacterial infection.

Bacterial pathogens	Bacterial effectors	Targeted pathway	Mitophagy	In vitro/in vivo study model	Mechanisms	Infection outcomes	Ref.
*S. aureus*	–	HDAC11	↑	BMDMs	Upregulates IL-10 and H3K9 acetylation; reduces mtROS	Bacterial survival ↑	^62^
	EV components	Parkin/apoptosis	↑	MAC-T, EV	Increase Parkin translocation to mitochondria; increase LC3 and TOM20 colocalization	–	^63^
	–	MEK1-ERK1/2	↑	BMDMs	Activates ERK1/2; suppresses CHEK2 and mtROS	Bacterial survival ↑	^64^
			↑	OM-mice model	Elevates p-MEK1 signals		
*H. pylori*	CagA	Mitophagy/ inflammasome	↑	GES-1, AGS	Increases PINK1, Parkin, P62, LC3, VDAC1 and TOM20; Increased LC3 colocalization with mitochondria	–	^66^
			↑	C57BL/6 mice	Upregulates PINK1, Parkin, p62, and LC3		
*B. pseudomallei*	BipD	IMMT Ubiquitination	↑	HEK293T, RAW264.7, PMs	BipD interacts with KLHL9 and KLHL13 recruiting CUL3; K63-linked ubiquitination of IMMT K211	Bacterial survival ↑	^67^
			↑	BALB/c mice	CUL3-mediated mitophagy		
*N. gonorrhoeae*	PorB	PINK/P62	↑	HeLa, HEK293T, HCT116, OMVs	OPTN and NDP52 recruitment; RNF213-dependent K63-linked polyubiquitination of PorB	Bacterial survival ↑	^68^
*S*. Typhimurium	SseJ	PHB2-PINK1/Parkin	↑	RAW264.7	Degrades MFN2 and COX4; increases TIMM23 and lysosome colocalization	Bacterial survival ↑	^69^
			↑	In vivo mouse model	Activation of PINK1 and PGAM		
*K. quasipneumoniae*	–	PINK1	↑	RAW264.7	Accumulates PINK1/ Parkin; Reduces MMP; Elevates LC3B and TGFβ1	–	^71^
			↑	Pulmonary fibrosis	Enhanced mtROS and TGF-β1		
*L. monocytogenes*	LLO	NLRX1	↑	PMs, 293T	Reduces mtROS levels	Bacterial survival ↑	^73^
			↑	WT and *Nlrx*1^–/–^ in vivo mice	NLRX1 oligomerization promotes LC3 binding via its LIR motif		
*M. tuberculosis*	–	BNIP3	↑	BMDMs/ RAW264.7	HIF-1α activation; reduction of MMP; lowers mtROS	Bacterial survival ↑	^75^
			↑	C57BL/6 mice	HIF-1α activation		
*B. abortus*	–	BNIP3L	↑	HeLa, iBMDMs	Iron-dependent HIF-1α activation upregulates BNIP3L, which further interacts with LC3	Bacterial egress ↑	^76^
*P. gingivalis*	–	PINK1	↓	BMDMs, patient’s tissue	Decreases PINK1, Parkin and LC3B expression; Increases mtROS	Progression of periodontitis	^78^
	–	PINK1	↑	HAECs	Upregulates PINK1 and Parkin; Decreases MMP; Increases LC3-II and p62 levels; Impaired xenophagy	Bacterial survival ↑	^79^

*BMDM* bone marrow-derived macrophages, *CUL3* cullin3, *EVs* extracellular vesicles, *GES-1* human gastric epithelial cell, *HAECs* human aortic endothelial cells, *HCT116* human colorectal carcinoma cell, *HEK* human embryonic kidney, *KLH* kelch-like family, *LLO* listerolysin O, *MAC-T* bovine mammary alveolar cell, *MMP* mitochondrial membrane potential, *mTOR* mammalian target of rapamycin, *OMV* outer membrane vesicle, *OM* osteomyelitis, *PM* peritoneal macrophage, *TGFβ1* transforming growth factor beta 1, *TIMM23* Translocase of Inner Mitochondrial Membrane 23, *TNF* tumor necrosis factor.

Collectively, the activation of mitophagy and mitophagy-regulating components contributes to the pathogenesis of various bacterial infections, with certain exceptions. However, the underlying mechanisms remain unclear, and future studies are required to elucidate the detailed molecular mechanisms underlying the pathogenesis of mitophagy-related infections in terms of individual bacterial species. Furthermore, comprehensive in vivo studies are required to identify novel therapeutic targets that modulate mitophagy.

## Mitophagy in viral infection

Mitophagy plays a dual role in promoting or evading host immune defense during various viral infections. In this section, we briefly discuss current knowledge regarding viral pathogenesis regulation by mitophagy and highlight several strategies by which different viruses evade host-protective mitophagy and instead co-opt mitophagy to support viral persistence and replication in distinct viral and cellular contexts.

### Protective mitophagy and viral escape

Mitophagy plays a protective role in the pathology of various viral infections. For example, Parkin- and BNIP3-mediated mitophagy is activated in mice with viral myocarditis and neonatal rat cardiac myocytes infected with coxsackievirus B3, playing a protective role against pathologies during viral myocarditis by maintaining cardiac function, whereas silencing Parkin aggravates viral myocarditis-induced apoptotic effects^80^. In addition, mitophagy activation through PINK1 suppresses Zika virus replication, suggesting a protective role of ubiquitin-dependent mitophagy during Zika virus infection^81^. Treatment with the mitochondrial uncoupling agent niclosamide inhibits Zika virus replication by promoting PINK1/Parkin-mediated mitophagy and facilitating the removal of fragmented mitochondria in vitro and in vivo in a mouse infection model^81^.

A recent study has shown that herpes simplex virus 1 (HSV-1) protein ICP34.5 or unique short 11 (US11) suppresses Parkin-mediated mitophagy, which promotes HSV-1 infection and NF-κB-mediated neuroinflammation. Mechanistically, the HSV-1 protein ICP34.5 and US11 deregulate the EIF2S1–ATF axis, leading to the transcriptional repression of the *PRKN*. Notably, Parkin overexpression increased resistance to HSV-1 infection and inhibited the pathogenesis of herpes simplex encephalitis^82^. The HSV-1 protein ICP34.5 also inhibits autophagy by binding to Beclin 1 and suppressing autophagosome formation^83^. During early infection, the HSV-1 protein ICP0 evades host defenses by reducing the expression of the autophagy adaptor proteins p62 and OPTN^84^. HSV-1-mediated microglial activation is caused by defective mitophagy and increased mitochondrial damage^85^.

Severe acute respiratory syndrome coronavirus 2 (SARS-CoV-2) Spike 1 and receptor-binding domain proteins induce expression of the NLRP3 inflammasome and IL-18, which play critical roles in cardiopulmonary inflammation and fibrosis by inhibiting mitophagy and enhancing mtROS^86^. Augmenting mitophagy through carbonyl cyanide 3-chlorophenylhydrazone treatment or blocking mtROS suppresses the Spike 1-induced maturation of IL-18^86^, suggesting mitophagy activation as a new therapeutic agent against SARS-CoV-2-induced pathological inflammation. Another study showed that foot-and-mouth disease virus infection induced mitophagy, thereby limiting viral replication. In this context, host heat shock protein 60 acts as a proviral factor, and its targeted depletion triggers Parkin-dependent mitophagy by promoting Ca^2+^/calmodulin-dependent protein kinase II-mediated phosphorylation of DRP1 at Ser616, thereby facilitating its translocation to the mitochondria. These findings suggest that heat shock protein 60 is a potential target for restricting foot-and-mouth disease virus infection through the modulation of mitophagy^87^. Collectively, these findings suggest that targeting PINK1/Parkin-mediated mitophagy may help control viral infections and provide new insights into mitophagy-targeted therapeutics. However, how different viruses and their specific effectors have evolved to subvert the mechanisms of escape from protective mitophagy during pathogenesis remains unclear.

### Pathogenic function of mitophagy during viral infection

Several recent studies have shown that virus-induced mitophagy promotes viral pathogenesis by regulating MAVS-dependent antiviral responses. During SARS-CoV-2 infection, diverse autophagy- and mitophagy-related genes, including *ATG9A*, *OPTN* and *ATG7*, contribute to enhanced viral replication and suppressed IFN signaling. In particular, depletion of autophagy/mitophagy genes, such as *ATG7* or *ATG9A*, increases the expression of MAVS, melanoma differentiation-associated protein 5, and retinoic acid inducible gene I (RIG-I) proteins, whereas the SARS-CoV-2 open reading frame (ORF) 9b co-localizes with MAVS to suppress RIG-I-like receptor-mediated antiviral defenses^88^. Moreover, SARS-CoV-2 ORF10 translocates to mitochondria and suppresses MAVS function by modulating mitophagy, thereby facilitating evasion of the type I IFN-mediated innate immune system^89^. In addition, the nucleoprotein of severe fever with thrombocytopenia syndrome virus induces mitophagy by translocating to mitochondria and interacting with Tu translation elongation factor, mitochondrial (TUFM), leading to MAVS degradation and suppression of antiviral signaling^90^. Furthermore, glycoprotein E of the varicella zoster virus promotes DRP1-dependent mitochondrial fission and PINK1/Parkin-mediated mitophagy, thereby inhibiting MAVS oligomerization and STING translocation, ultimately impairing MAVS- and STING-dependent antiviral IFN responses^91^.

The coronavirus matrix (M) protein activates mitophagy by recruiting 3-phosphoinositide-dependent protein kinase-1, which redirects p62 binding from the M protein to the mitochondria, thereby suppressing virophagy and antiviral innate immunity^92^. Notably, the 3-phosphoinositide-dependent protein kinase-1-targeting peptide induces antiviral innate immune responses through enhanced virophagy and confers protective effects in mouse infection models^92^. Senecavirus A, an emerging picornavirus associated with swine diseases, drives viral pathogenesis by inducing mitophagy through the direct interaction of viral protein 2C with K27-linked ubiquitinated TUFM, thereby facilitating viral replication^93^. In addition, nonstructural protein 1 of respiratory syncytial virus functions as a mitophagy receptor by binding to LC3B and the mitochondrial protein TUFM, thereby inducing mitophagy, promoting viral replication and suppressing virus-induced IFN responses^94^. Furthermore, hepatitis C virus NS5A, an essential regulator of the viral life cycle, induces mitophagy by recruiting the mitophagy receptors NDP52 and OPTN to mitochondria^95^. However, the precise role of hepatitis C virus NS5A-induced mitophagy in liver disease pathogenesis remains to be elucidated.

Pseudorabies virus, also known as suid herpesvirus 1, causes a highly contagious disease that primarily affects swine^96,97^. Pseudorabies virus infection disrupts IFN-I production by modulating mitochondrial homeostasis through PINK1/Parkin-mediated mitophagy^98^. Impaired IFN-I production promotes viral replication and suppresses antiviral innate immune signaling^98^. During infection with human herpesvirus 8, also known as Kaposi’s sarcoma-associated herpesvirus, viral IFN regulatory factor 1 interacts with GABA type A receptor-associated protein like 1, a member of the mammalian ATG8 family, thereby inducing mitophagy and promoting productive viral replication. In this process, the mitophagy receptor BNIP3L is required for viral IFN regulatory factor 1 aggregation, stabilization, and interaction with GABA type A receptor-associated protein like 1^99^. These studies suggest that viral effectors can function as mediators of mitophagy to promote replication and pathogenesis.

The influenza A virus polymerase basic protein 1 frame 2 (PB1-F2) protein inhibits MAVS by translocating to mitochondria via Tom40 channels^100^. PB1-F2-induced loss of mitochondrial membrane potential contributes to mitochondrial fragmentation^100^. In addition, PB1-F2 directly represses MAVS-associated innate immune signaling and interacts with TUFM and LC3B via an LIR motif, thereby inducing TUFM-dependent mitophagy^101^. By contrast, the nucleoprotein of influenza A virus promotes mitophagy through interaction with MAVS and toll-interacting protein, leading to attenuation of innate immunity and enhanced viral replication^102^. Furthermore, primary influenza A virus infection, followed by secondary bacterial infection, may contribute to pathogen proliferation and inflammation via activation of the PINK1/Parkin pathway^103^.

Infection with swine arterivirus, such as porcine reproductive and respiratory syndrome virus, triggers mitochondrial fission and mitophagy, thereby promoting aerobic glycolysis and facilitating replication. Porcine reproductive and respiratory syndrome virus-induced increases in mitochondrial Ca^2+^, derived from the ER, activate the Ca^2+^/calmodulin-dependent protein kinase β–AMP-activated protein kinase–DRP1 signaling pathway, further inducing mitophagy via the PINK1/Parkin pathway^104^. African swine fever virus protein p17 induces mitophagy by promoting the interaction between p62 and translocase of outer mitochondrial membrane 70, thereby facilitating MAVS degradation and suppressing proinflammatory cytokine production^105^. Furthermore, porcine epidemic diarrhea virus infection activates mitophagy through interaction with tripartite motif containing 28, thereby inhibiting the Janus kinase (JAK)–STAT1 pathway and promoting viral replication^106^.

In classical swine fever virus (CSFV) infection, a highly contagious disease in pigs, elevated lactate dehydrogenase A expression and increased lactate levels suppress PINK1/Parkin-mediated mitophagy. In addition, lactate activates the JAK1–STAT1–IFN-stimulated gene 15 pathway and reduces CSFV replication by inhibiting carbonyl cyanide 3-chlorophenylhydrazone-induced mitophagy^107^. Moreover, CSFV-mediated inhibition of lactate dehydrogenase B regulates mitophagy, which provides a favorable environment for pathogen proliferation by inhibiting apoptosis^108^. Notably, CSFV nonstructural proteins, including NS3, NS4B and NS5A, interact with the endosomal sorting complexes required for transport machinery, including CHMP1A, CHMP1B, CHMP4B and VPS4A, thereby contributing to phagophore closure and mediating CSFV-induced mitophagy^109^.

Mitophagy is also involved in the pathogenesis of Alongshan virus—a newly discovered virus of the Flaviviridae family. The nonstructural protein NSP1 interacts with and degrades STAT2, leading to disrupted mitochondrial dynamics, mitophagy activation, suppressed mitochondrial biogenesis and suppression of innate immune responses^110^. Strategies for inhibiting mitophagy and promoting mitochondrial biogenesis promote host defense by counteracting NSP1-mediated suppression of the innate immune response during Alongshan virus infection^110^. Prototype foamy virus, a retrovirus that causes latent infection, promotes mtROS generation and PINK1/Parkin-dependent mitophagy^111^. However, the consequences of mitophagy modulation by prototype foamy virus on viral pathogenesis remain unclear.

The Newcastle disease virus is known for oncolytic activity and tumor-selective replication behavior, which suggests promise as a viral therapeutic targeting cancer cells^112–114^. Newcastle disease virus reprograms cell metabolism by inducing mitochondrial damage, glycolysis and mitochondrial shift from fusion to fission. Imbalanced AMP, ADP and ATP levels contribute to activation of the AMP-activated protein kinase and inhibition of mammalian target of rapamycin, leading to a subsequent decrease in mitochondrial Sirtuin 3 (SIRT3) via PINK1/Parkin-mediated mitophagy, thereby supporting viral replication^115^. In the case of enterovirus 71 infection, which is associated with severe and often fatal disease in young infants, the virus triggers PINK1/Parkin-mediated mitophagy, leading to degradation of MAVS and the release of mitochondria containing enterovirus 71 virions into the extracellular space, thereby facilitating viral replication and dissemination^116^.

Together, these findings offer an improved understanding of how viruses manipulate or co-opt mitophagy to support their pathogenesis and highlight opportunities for developing promising therapeutic targets against specific viral infections. The mechanisms by which different viruses evade host-protective mitophagy and contribute to viral pathogenesis are summarized in Table 2. Future studies are needed to elucidate the precise molecular mechanisms that characterize the distinct virulence factors that exploit host defenses and contribute to pathogenesis.

**Table 2:** Roles and mechanisms of mitophagy in viral infection.

Virus	Effector	Targeted pathway	In vitro/in vivo study model	Mechanisms	Infection outcomes	Ref.
Upregulated mitophagy						
ALSV	NSP1	STAT2	HEK293T, HepG2, A549, Raw264.7 and STAT2^−/−^ Vero cell lines	NSP1 degrades STAT2 to suppress innate immune response	Viral replication ↑	^110^
ASFV	p17	SQSTM1; TOMM70	HEK293T, Vero and 3D4121 cell lines	p17 promotes mitophagy by facilitating the interaction of p62 with TOMM70	Viral replication ↑	^105^
		MAVS	HEK293T, Vero and 3D4121 cell lines	p17 facilitates MAVS degradation via the mitophagic pathway to suppress innate immunity	Viral replication ↑	^105^
CoV	M protein	PDPK1; p62	PDPK1^*fl/fl*^ and PDPK-cKO mice and SQSTM1 KO mice; HEK293T, Vero E6, DF-1 and MDCK cell lines	CoV M protein recruits PDPK1 to redirect p62 binding from M protein to mitochondria; suppresses virophagy	Viral replication ↑	^92^
CSFV	NS3	LDHB	Piglets; PK-15, HEK293T and 3D4/2 cell lines	NS3 inhibits LDHB to induce mitochondrial fission and mitophagy	Viral replication ↑	^108^
	NS3, NS4B, NS5A	ESCRT machinery	PK-15 cell line	NS3, NS4B and NS5A promote phagophore sealing during mitophagy	Viral replication ↑	^109^
CVB3	–	Parkin; BNIP3	WT Balb/c; NRCMs	CVB3 activates Parkin/BNIP3-mediated mitophagy with blockade of autophagic flux	Viral replication ↑; cardiac dysfunction	^80^
EV71	–	PINK1/Parkin; MAVS	HeLa cell line	EV71 activates PINK1/Parkin-mediated mitophagy; degrades MAVS	Viral replication ↑	^116^
FMDV	–	PINK1/Parkin; DRP1	PK-15 cell line	FMDV activates PINK1/Parkin-dependent mitophagy via CaMKII-mediated DRP1 phosphorylation	Viral replication ↑	^87^
		HSP60	PK-15 cell line	FMDV depletes host factor HSP60 to promote DRP1 mitochondrial translocation	Viral replication ↑	^87^
HCV	NS5A	PINK1/Parkin	Huh7 and Huh7/RFP-Parkin/Mito-GFP cell lines	NS5A induces PINK1 stabilization and Parkin translocation	Viral replication ↑	^95^
		NDP52; OPTN	Huh7 and Huh7/NDP52 KO cell lines	NS5A recruits mitophagy receptors NDP52 and OPTN	Viral replication ↑	^95^
HHV-8 / KSHV	vIRF-1	BNIP3L; TUFM; GABARAPL1	HEK293T, HeLa.Kyoto, iSCK and iBCBL cell lines	vIRF-1 interacts with BNIP3L and TUFM; Binds directly to GABARAPL1; vIRF-1 induces BNIP3L oligomerization; vIRF-1 promotes recruitment of autophagosomal membranes to mitochondria	Viral replication ↑	^99^
IAV	PB1-F2	MAVS; TUFM; LC3B	HEK293, TUFM-KO HEK293T, HeLa, A549, TUFM-KO A549, J774A.1, BMDMs and MDCK cell lines	PB1-F2 links TUFM and LC3B to induce MAVS degradation	Viral replication ↑	^100,101^
	NP	MAVS; TOLLIP	MDCK, HEK293T, U2OS, A549, *TOLLIP*^−/−^, *ATG7*^−/−^, *ATG12*^−/−^, *MAV*^−/−^, *NBR1*^−/−^ and *CALCOCO2*^−/−^ cell lines	NP recruits TOLLIP and MAVS to autophagosomes	Viral replication ↑	^102^
	NS1	ULK1; BNIP3	A549 and MDCK cell lines	NS1 enhances mitophagy via ULK1/BNIP3	Viral replication ↑	^103^
	M2	–	A549 and MDCK cell lines	M2 blocks autophagosome–lysosome fusion; M2-mediated mtDNA release triggers antiviral response	Viral replication ↑	
NDV	–	PINK1/Parkin; SIRT3	HeLa, *PINK1*^−/−^ HeLa, *HIF1A*^−/−^ HeLa, HEK293, *ATG7*^−/−^HEK293, A549, HepG2, Df-1 cell lines and CEF primary cell	NDV degrade mitochondrial SIRT3 via PINK1/Parkin pathway; reprogram metabolism	Viral replication ↑	^115^
PEDV	N protein	TRIM28; JAK-STAT1	Vero E6 and IPEC-J2 cell lines	N protein interacts with TRIM28 to induce mitophagy; inhibits JAK-STAT1 and IFN-β signaling	Viral replication ↑	^106^
PFV	Gag	PINK1/Parkin; Rab5a; p62	HEK293T and HT1080 cell lines	Gag induces mitochondrial damage and mtROS; Gag upregulates Rab5a; Gag activates PINK1-Parkin pathway; Gag facilitates translocation of LC3 and p62 to mitochondria	–	^111^
PRRSV	–	PINK1/Parkin	Marc-145 cell line and PAMs	PRRSV induces mitochondrial fission; promotes aerobic glycolysis	Viral replication ↑	^104^
PRV	–	PINK1/Parkin; MAVS	PK-15 cell lines	PRV triggers PINK1-Parkin-mediated mitophagy; promotes MAVS degradation	Viral replication ↑	^98^
RSV	NS1	LC3B; TUFM; DRP1	WT Balb/c, TUFM-KO mice, ATG5^wt^ and ATG5^ko^ mice; Vero E6, HEK293, Hep-2 and HeLa cell lines	NS1 interacts with TUFM and LC3B; NS1 induces DRP1-dependent mitochondrial fission; NS1 suppresses RIG-I response	Viral replication; Lung pathology ↑	^94^
SARS-CoV-2	ORF9b; ORF10	MAVS	HEK293T, HeLa-ACE2 stable and BNIP3L-KO cell lines	ORF9b and ORF10 induce MAVS degradation via mitophagy	Viral replication ↑	^89^
SFTSV	Nucleoprotein	TUFM; MAVS	HEK293T and HeLa cell lines	Nucleoprotein translocates to mitochondria and interacts with TUFM; nucleoprotein binds LC3 via N-terminal LIR motif; nucleoprotein leads to MAVS degradation	Viral replication ↑	^90^
SVA	2C protein; VP2; VP3; 3A	TUFM; p62	BHK-21, PK-15, IBRS-2, ST and HEK293T cell lines	2C, VP2, VP3 and 3A proteins recruit SQSTM1 to link ubiquitinated TUFM to LC3	Viral replication ↑	^93^
VZV	gE	PINK1/Parkin; DRP1; MAVS; STING	HeLa, HEK293T, MRC5 and HeCaT cell lines	gE promotes DRP1-mediated fission; Interacts with LC3; gE upregulates mtROS to activate mitophagy; gE sequesters MAVS and blocks STING translocation	Viral replication ↑	^91^
ZIKV	–	PINK1	WT balb/c; A549 cell line	ZIKV activates PINK1-mediated mitophagy to eliminate damaged mitochondria	Viral replication ↓	^81^
Downregulated mitophagy						
CSFV	–	PINK1/Parkin; LDHA	Piglets; PK-15, 3D4/2 cell lines	CSFV suppresses mitophagy to activate JAK1–STAT1-ISG15	Viral replication ↓	^107^
HSV-1	ICP34.5; US11	Beclin 1; cGAS–STING	Balb/c and C57BL/6; PKR^−/−^ and PKR^wt^ mice; N2a, Vero, HMC3, HEK293, MCF7, SK-N-SH, Beclin^−/−^ and Beclin^+/+^ ES cell lines	ICP34.5 and US11 bind Beclin 1; ICP34.5 and US11 blocks mitophagic flux to enhance cGAS–STING signaling	Viral replication ↑; Neuroinflammation ↑	^82,83^
	ICP0	PINK1/Parkin; p62; OPTN	HEL, Vero, HEK293, Hep-2, HMC3, THP-1 and SK-N-SH cell lines	ICP0 triggers proteasomal degradation of p62 and OPTN	Viral replication ↑	^84^
SARS-CoV-2	Spike 1; RBD	–	Patient lung tissue; C57BL/6J hACE2 transgenic mice	Spike 1 and RBD inhibit mitophagy and increase mtROS	Viral replication ↑	^86^

*2C/3A* viral protein 2C/3A, *ALSV* Alongshan virus, *ASFV* African swine fever virus, *ATG5KO* autophagy-related 5 knockout, *CaMKII* calcium/calmodulin-dependent protein kinase II, *CoV* coronavirus *CVB3* Coxsackievirus B3, *ESCRT* endosomal sorting complexes required for transport, *EV71* enterovirus 71, *FMDV* foot-and-mouth disease virus, *GABARAPL1* GABA type A receptor-associated protein-like 1, *Gag* Groupspecific antigen, *HCV* Hepatitis C virus, *HHV-8 / KSHV* Human herpesvirus 8 / Kaposi’s sarcoma-associated herpesvirus, *HSP60* Heat shock protein 60, *IAV* influenza A virus, *ICP0/ICP34.5* infected cell protein 0/34.5, *IFN-β* interferon beta, *ISG15* interferon-stimulated gene 15, *LDHB* lactate dehydrogenase B, *NDV* Newcastle disease virus, *NP* nucleoprotein, *N* protein nucleocapsid protein, *NS1/NS3/NS4B/NS5A* nonstructural protein 1/3/4B/5A, *PAMs* porcine alveolar macrophages, *Parkin* E3 ubiquitin-protein ligase parkin, *PDPK13*-phosphoinositide dependent protein kinase 1, *PEDV* porcine epidemic diarrhea virus, *PFV* prototype foamy virus, *PRRSV* porcine reproductive and respiratory syndrome virus, *PRV* pseudorabies virus, *RBD* receptor-binding domain, *RSV* respiratory syncytial virus, *SFTSV* severe fever with thrombocytopenia syndrome virus, *Spike 1 (S1)* Spike glycoprotein subunit 1, *SVA* Senecavirus A, *TOLLIP* Toll interacting protein, *TRIM28* tripartite motif containing 28, *vIRF-1* viral interferon regulatory factor 1, *VP2/VP3* viral protein 2/3, *VZV* Varicella zoster virus, *ZIKV* Zika virus.

## Mitophagy in sepsis

Mitophagy typically exerts dual functions: protective functions in sepsis or sepsis-related pathologies. For example, PINK1 protected dendritic cell function in a cecal ligation puncture model of sepsis by maintaining mitochondrial quality control^117^. PINK1 deficiency led to increased mitochondrial fission through DRP1 and induced dendritic cell dysfunction and apoptosis in mice undergoing cecal ligation puncture^117^. A recent study showed that transmembrane BAX inhibitor motif containing 1 (TMBIM1), a stress-responsive protein, is elevated in the myocardium during lipopolysaccharide (LPS) challenge and plays a cardioprotective role by interacting with Parkin, facilitating mitophagy activation^118^. Similarly, transmembrane BAX inhibitor motif containing 6, also known as BAX inhibitor-1 (TMBIM6/BI-1), prevents BAX activation, translocation to the mitochondria, and apoptosis in cells^119^. Moreover, TMBIM6 overexpression blocks VDAC1 oligomerization, thereby improving the mitochondrial quality control system and alleviating sepsis-related myocardial injury^120^.

The mitophagy receptor FUNDC1 ameliorated septic cardiomyopathy by interacting with and suppressing the lipid peroxidation enzyme acyl-CoA synthetase long-chain family member 4 via ferroptosis regulation^121^. Additional bioinformatics analysis identified several clusters by mitophagy-related typing of 348 sepsis samples and showed that the cluster with the highest degree of mitophagy was associated with the lowest disease severity^122^. Notably, prohibitin 1 (PHB1) was the most differentially expressed mitophagy gene among the clusters and was negatively correlated with sepsis severity^122^. PHB1 forms a hetero-oligomeric complex with PHB2; unlike PHB2, which directly functions as a mitophagy receptor through its LIR domain, PHB1 lacks an LIR domain and regulates mitophagy indirectly through structural scaffolding and modulation of the process. Another study showed that PHB1 silencing increases mtDNA levels in the cytoplasm and triggers NLRP3 inflammasome activation^122^. In addition, Notch1 expression in cardiac macrophages of septic murine models modulates cardiac injury through mammalian STE20-like kinase 1 expression. Notch1-knockout models show improved cardiac performance, restoring impaired mitophagy and preventing activation of the NLRP3 inflammasome caused by sepsis^123^. Moreover, the endosomal adaptor protein APPL1 interaction with Ras-related protein Rab-5A (Rab5) facilitates mitophagy, thereby conferring the protection of mice against endotoxin-induced sepsis^124^. Together, these studies suggest that targeted activation of mitophagy pathway proteins can improve sepsis pathology and disease severity.

Numerous mitophagy-inducing molecules exhibit protective functions that improve survival and ameliorate pathology in sepsis models. For example, sirtuins (SIRTs), the NAD^+^-dependent HDACs, ameliorate sepsis and related pathologies. A recent study showed that SIRT1 deficiency exacerbates sepsis-induced acute lung injury (ALI). SIRT1 induces mitophagy by interacting with the late endosome Ras-related protein Rab-7A (Rab7), thereby suppressing excessive activation of the STING and NLRP3 inflammasome pathways. Moreover, pharmacological activation of SIRT1 with SRT1720 or overexpression of SIRT1 exerted protective effects against sepsis-induced ALI^125^. In addition, SIRT3, a protein deacetylase, controls sepsis-induced ALI through Parkin-dependent mitophagy. SIRT3 deficiency leads to impaired mitophagy through Parkin suppression, aggravated mtROS generation, cytosolic release of mtDNA and activation of the NLRP3 inflammasome and pyroptosis in lung endothelial cells^126^. Moreover, the neurohormone melatonin induces mitophagy through SIRT3-mediated deacetylation of mitochondrial transcription factor A. This leads to attenuation of sepsis-induced acute kidney injury (AKI)^127^. Endothelial–mesenchymal transition is involved in sepsis-induced myocardial injury, which leads to defective mitophagy. Modulating SIRT3 attenuates sepsis-induced myocardial injury via SIRT3-mediated deacetylation of the PINK1/Parkin pathway, leading to mitophagy upregulation^128^.

Runt-related transcription factor 1, which is involved in development and hematopoiesis, improves ALI by controlling NF-κB signaling pathway in epithelial cells^129^. A recent study revealed that this mechanism depends on mitophagy activation, at least partially through the transcriptional regulation of mitophagy adaptor proteins such as p62 and BNIP3L^130^. In addition, dual-specificity phosphatase 1 interacts with valosin-containing protein and suppresses valosin-containing protein phosphorylation at Ser784 in response to LPS. This action preserves mitochondrial quality control and improves mitophagy, thereby reducing pathological events during endotoxemia-induced myocardial injury^131^. HDAC3 deficiency also improves mitochondrial quality control and fusion and decreases mitophagy, thereby alleviating epithelial damage and sepsis-induced ALI^132^. The direct roles of dual-specificity phosphatase 1 and HDAC3 in regulating mitophagy are unclear, but these findings suggest that mitochondrial quality control, through mitophagy activation, can attenuate sepsis and related pathologies.

At the onset of sepsis, type 3 deiodinase inactivates thyroid hormones, leading to disruption of mitophagy and contributing to anabolic resistance, whereas sepsis models with type 3 deiodinase knockdown show a reduction in sepsis-induced muscle wasting via enhanced mitophagy^133^. Moreover, several molecules are involved in mitophagy suppression and impairment, thereby promoting sepsis. PGAM5 causes mitochondrial impairment by interacting with BNIP3^134^. PGAM5-mediated mitophagy dysfunction is crucial for promoting sepsis-induced microvascular injury and inflammatory damage^135^. Moreover, insulin-like growth factor-binding protein 7 promotes inflammation-coupling tubular damage and aggravates septic AKI by suppressing BNIP3/BNIP3L-dependent mitophagy^136^. These data strongly suggest that targeting either insulin-like growth factor-binding protein 7 or PGAM5 is beneficial for the development of novel therapeutics against sepsis-associated diseases to preserve mitochondrial homeostasis and clearance. In polymicrobial sepsis, PINK1/Parkin-mediated mitophagy suppresses macrophage activation, thereby impairing bacterial clearance and reducing mouse survival^137^. The roles and mechanisms by which mitophagy regulates sepsis are summarized in Table 3. Further studies are needed to determine the precise role of mitophagy in complex disease pathology, with the aim of reducing hyperinflammation while enhancing antimicrobial activity.

**Table 3:** The roles and mechanisms of mitophagy regulators in sepsis model.

Regulators	In vitro/in vivo study model	Mechanisms	Outcomes	Ref
Host protective				
PINK1	LPS-stimulated BMDCs	PINK1 recruits Parkin and activates mitophagy	Loss of dendritic cell function and increased apoptosis in PINK1 deficiency	^117^
	CLP mice	PINK1 maintains DC function and decreases DC apoptosis	Exacerbated sepsis and increased mortality in PINK1 deficiency	
TMBIM1	LPS-stimulated cardiomyocytes	TMBIM1 interacts with Parkin and promotes its translocation to damaged mitochondria	Improvement of LPS-induced mitochondrial dysfunction in TMBIM1-overexpressing cardiomyocytes	^118^
	LPS-injected mice	TMBIM1 enhances Parkin-mediated mitophagy and preserves mitochondrial quality in septic hearts	Decreased mortality following LPS challenge in TMBIM1-overexpressing mice	
TMBIM6	Primary mouse cardiomyocytes and HL-1 mouse cardiac muscle cells	TMBIM6 binds to VDAC1, prevents its oligomerization, and maintains mitochondrial Ca²⁺ homeostasis	Mitochondrial and cardiac dysfunction in TMBIM6 deficiency	^120^
	LPS-injected mice	TMBIM6 preserves mitochondrial Ca²⁺ homeostasis and MQC		
FUNDC1	LPS-stimulated mouse cardiomyocytes	FUNDC1 interacts with ACSL4 and reduces ACSL4-dependent ferroptosis	Reduction of contractile function and disruption of mitochondrial homeostasis under FUNDC1 deficiency	^121^
	CLP mice	FUNDC1 maintains mitophagy, suppresses ACSL4-associated ferroptosis, and preserved mitochondrial integrity and cardiac function	Exacerbation of all CLP-induced myocardial injuries in FUNDC1-deficient mice	
PHB1	Inflammasome-induced primary mouse PMs	PHB1 promotes LC3-dependent mitophagy and suppresses NLRP3 inflammasome	Poor sepsis prognosis associated with low PHB1 expression	^122^
Notch1	Inflammasome-induced primary mouse BMDMs and RAW264.7	Notch1 inhibits mitophagy through promoting *Mst1* transcription	Decreased cardiomyocyte apoptosis under Notch1 deficiency	^123^
	LPS-injected mice	–	Improved survival in Notch1-deficient mice after LPS challenge	
APPL1	Inflammasome-induced BMDMs	APPL1 interacts with Rab5 and translocates to damaged mitochondria to facilitate mitophagy	Inflammasome hyperactivation in APPL1 deficiency	^124^
	LPS-injected mice	–	APPL1 deficiency-induced metabolic dysfunction characterized by weight loss, hypothermia, glucose intolerance and insulin resistance	
SIRT1	LPS-stimulated primary mouse lung vascular ECs	SIRT1 inhibits NLRP3/STING activation through inducing Rab7-mediated mitophagy	Disruption of the lung endothelial barrier under SIRT1 deficiency	^125^
	CLP mice	SIRT1 maintains mitophagic flux and suppresses NLRP3/STING signaling in lung tissue	Excessive lung injury, vascular leakage and inflammation; reduced survival under SIRT1-suppressed condition	
SIRT3	LPS-stimulated primary mouse lung vascular ECs	SIRT3 acts upstream of Parkin and maintains Parkin-mediated mitophagy	Increased endothelial pyroptosis and endothelial barrier integrity damage under SIRT3 deficiency	^126^
	CLP mice	SIRT3 suppresses endothelial pyroptosis through maintaining Parkin-dependent mitophagy during septic ALI	Increased lung vascular leakage, activated lung endothelial pyroptosis, and reduced survival under SIRT3-suppressed condition	
	LPS-stimulated HK-2 cells	SIRT3 promotes mitophagy through enhancing TFAM deacetylation	Preservation of renal tubular epithelial cell viability under melatonin-treated condition	^127^
	CLP mice treated with melatonin	–	Decreased inflammation, inhibition of SAKI progression, increased survival under melatonin-treated condition	
RUNX1	CCCP-treated A549 cells	RUNX1 promotes mitophagy through transcriptional activation of P62 and BNIP3L	Impaired clearance of damaged mitochondria under RUNX1 deficiency	^130^
	LPS-injected mice	RUNX1 increases p62 and BNIP3L expression, reduces mitochondrial damage, and suppresses cGAS–STING/NLRP3	Alleviated LPS-induced lung injury by RUNX1-dependent mitophagy activation	
DUSP1	LPS-stimulated HL-1 cardiomyocytes	DUSP1 interacts with VCP and suppresses VCP Ser784 phosphorylation, thereby preserving mitophagy	Increased inflammatory cytokines, injury markers and apoptosis suppressed by DUSP1	^131^
	LPS-injected mice	DUSP1 suppresses VCP Ser784 phosphorylation and restores MQC in endotoxemic hearts	Elevated cardiac injury markers suppressed by DUSP1	
IGFBP-7	Primary mouse RTECs and Human HK-2 cells	IGFBP-7 suppresses BNIP3L/BNIP3-dependent mitophagy through IGF1R deactivation	Exacerbation of mitochondrial dysfunction and augmentation of caspase-mediated apoptosis by IGFBP-7	^136^
	CLP mice	IGFBP-7 suppresses BNIP3L/BNIP3-dependent mitophagy, thereby promoting inflammation-coupled tubular injury and septic AKI progression	Reduction of survival rate and exacerbation of septic AKI severity in the presence of IGFBP-7	
PGAM5	LPS-stimulated coronary microvascular endothelial cells	PGAM5 suppresses PINK1/Parkin-mediated mitophagy through VDAC1, resulting in endothelial apoptosis	Exacerbation of mitochondrial dysfunction and mitochondrial apoptosis under PGAM5-upregulated condition	^135^
	LPS-injected mice	PGAM5 suppresses PINK1/Parkin-dependent mitophagy through VDAC1, leading to septic injury	Recovery of cardiac ejection function and decreased apoptosis in PGAM5-knockout mice	
Host detrimental				
HDAC3	LPS-stimulated primary mouse AT2 cells	HDAC3 deacetylates FOXO1, promotes FOXO1-dependent ROCK1 transcription and disrupts MQC	HDAC3-induced mitophagy activation associated with AT2 apoptosis and epithelial barrier disruption	^132^
	LPS-injected AT2-HDAC3 cKO mice	–	Attenuation of lung injury, preservation of epithelial barrier integrity and improvement in survival	
STAT1	RAW 264.7, mouse BMDMs and mouse PMs	STAT1 suppresses PINK1-dependent mitophagy through caspase-1/11 activation	Promotion of classical macrophage activation and enhancement of bactericidal clearance with mitophagy inhibition	^137^
	LPS-injected mice model and CLP mice	STAT1 promotes mitophagy inhibition, leading to macrophage activation, enhanced bacterial clearance and improved survival during sepsis	Reduction of bacterial load and improvement of survival during polymicrobial sepsis with mitophagy inhibition	

*ACSL4* acyl-CoA synthetase long-chain family member 4, AM alveolar macrophage, *APPL1* adaptor protein phosphotyrosine interacting with PH domain and leucine zipper 1, *AT2* cells, alveolar type II epithelial cells, *BMDCs* bone marrow-derived dendritic cells, *BMDMs* bone marrow-derived macrophages, *CCCP* carbonyl cyanide 3-chlorophenylhydrazone, *CLP* cecal ligation and puncture, *DCs* dendritic cells, *DUSP1* dual-specificity phosphatase 1, ECs, endothelial cells *FOXO1* forkhead box protein O1, HL-1 mouse atrial cardiomyocyte cell line, HK-2 human kidney proximal tubular epithelial cells, *IGF1R* insulin-like growth factor 1 receptor, IGFBP-7 insulin-like growth factor binding protein 7, *MQC* mitochondrial quality control, Mst1 mammalian sterile 20-like kinase 1, PMs peritoneal macrophages, *ROCK1* Rho-associated coiled-coil containing protein kinase 1, *RUNX1* runt-related transcription factor 1, *RTECs* renal tubular epithelial cells, *SAKI* sepsis-associated acute kidney injury, *TFAM* mitochondrial transcription factor A, *VCP* valosin-containing protein.

## Therapeutic potential of mitophagy modulation

In this section, we discuss current experimental approaches using therapeutic agents to modulate and target infection and inflammation.

### Targeting mitophagy for antiviral responses

A recent study showed that a novel mitophagy activator, ALT001, suppresses mitochondrial damage caused by HSV-1 infection by activating mitophagy via the ULK1/Rab9-mediated pathway. Notably, ALT001-induced ULK1/Rab9-dependent mitophagy promotes IFN-mediated antiviral immune function against HSV-1 infection and ameliorates HSV-1-triggered neuroinflammation^138^. Furthermore, gut microbial metabolites have emerged as regulators of mitophagy activation, thereby inducing host protection. The gut microbial metabolite nicotinamide N-oxide restores NAD^+^-dependent mitophagy, restricting microglial activation and disease progression in herpes simplex encephalitis^85^. Similarly, taurine, another gut microbial metabolite, activates Parkin expression to stimulate mitophagy, thereby controlling HSV-1 infection and suggesting a novel therapeutic strategy for herpes simplex encephalitis^82^.

Several agents ameliorate viral infections by activating mitophagy. For example, the natural product Xijiao Dihuang Decoction combined with Yinqiao powder exhibits beneficial effects against influenza virus-induced pneumonia by inducing mitophagy to remove damaged mitochondria and ameliorating NLRP3 inflammasome activation and pyroptosis^139^. The arctigenin derivative TZOA is critical for maintaining mitochondrial homeostasis by suppressing virus-induced mitochondrial fragmentation and activating mitochondrial fusion and mitophagy during infection with infectious hematopoietic necrosis virus, a member of the genus Novirhabdovirus. This action favors the clearance of damaged mitochondria, which restores MAVS-mediated IFN expression and induces an antiviral response^140^. Verbenalin treatment shows protective effects in a coronavirus pneumonia model and in a mouse alveolar macrophage cell line through activation of PINK1/Parkin-mediated mitophagy, with concomitant attenuation of NLRP3 inflammasome activation and cell injury^141^.

By contrast, several reports have highlighted the beneficial effects of the pharmacological inhibition of mitophagy by mitochondrial division inhibitor-1 for regulating viral infection. For example, in SARS-CoV-2 infection, mitochondrial division inhibitor-1 treatment impacts immunometabolic remodeling by suppressing excessive glucose metabolism, thereby enhancing the generation of SARS-CoV-2-specific CD8^+^ cytotoxic T cells and augmenting memory cell proliferation^142^. A list of antiviral agents targeting mitophagy is summarized in Table 4.

**Table 4:** Antiviral agents targeting mitophagy.

Agent	Pathogen	Disease model	Target	Biological responses	Outcomes	Ref.
Taurine	HSV-1	HSE	Microglia	Enhances Parkin expression and activates Parkin-mediated mitophagy	Modulates HSV-1 infection and the NF-κB-mediated neuroinflammation.	^82^
Nicotinamide *N*-oxide	HSV-1	HSE	Microglia	NAD^+^-dependent mitophagy	Inhibits microglia activation and HSE progression	^85^
ALT001	HSV-1	AD	Microglia	ULK1/Rab9-mediated pathway	Reduce HSV-1-induced neuroinflammation	^138^
XDY	Influenza virus	Influenza pneumonia	Macrophage	Activates mitophagy-mediated ROS scavenging	Inhibits ROS/NLRP3/pyroptosis axis and ameliorates inflammation	^139^
TZOA	IHNV	Viral infection in oncorhynchus mykiss	Endothelial progenitor cell	Facilitates mitochondrial fusion and mitophagy	Restores mitochondrial homeostasis and MAVS-mediated IFN expression	^140^
Verbenalin	Coronavirus 229E	coronavirus pneumonia	Macrophage	Enhances PINK1/Parkin-mediated mitophagy	Reduces inflammation and lung injury	^141^
Mdivi-1	SARS-CoV-2	COVID-19	CD8^+^Tc	Inhibits mitophagy	Restores dysregulated glucose metabolism and normalizes CD8^+^Tc activity	^142^

*AD* Alzheimer’s disease, CD8^+^Tc CD8^+^ cytotoxic T cell, *COVID-19* coronavirus disease 2019, *HSE* herpes simplex encephalitis, *IHNV* infectious hematopoietic necrosis virus, *Mdivi-1* mitochondrial division inhibitor-1, *NAD*^+^ nicotinamide adenine dinucleotide, *XDY* Xijiao Dihuang Decoction combined with Yinqiao powder.

### Targeting mitophagy for antisepsis responses

Various reagents and small molecules control sepsis by activating mitophagy. In a model of acute respiratory distress syndrome, esketamine inhibits LPS-induced lung injury and inflammation by activating mitophagy via ULK1/FUNDC1 signaling^143^. Emodin, a bioactive anthraquinone extracted from *Rheum palmatum* (Radix Rhei) and *Polygonum cuspidatum* (Polygonaceae), inhibits NLRP3 inflammasome formation^144,145^. In sepsis models, emodin exerts a protective function via FUNDC1-mediated mitophagy^146^. Another recent study showed that high concentrations of hydrogen are beneficial for sepsis-associated encephalopathy by promoting PINK1/Parkin-mediated mitophagy, while suppressing the cGAS–STING–IFN regulatory factor 3 signaling pathway, thereby attenuating neuroinflammation^147^. In addition, bergapten—a furocoumarin phytohormone found in herbal medicines and fruits—contributed to the attenuation of tissue inflammation and injury in a mouse model of *Citrobacter rodentium*-induced intestinal inflammation and *Escherichia coli*-induced sepsis. Mechanistically, bergapten suppressed NLRP3 inflammasome activation and pyroptotic cell death in macrophages via mitophagy activation^148^. However, the underlying mechanisms remain unclear. Dendrobine, an alkaloid found in *Dendrobium nobile*, is a potent therapeutic agent for sepsis-associated AKI. Dendrobine decreases proinflammatory cytokines and upregulates mitophagy-related proteins such as PINK1, Parkin and LC3B/A, thereby enhancing mitophagy and mitigating sepsis-associated AKI^149^.

Puerarin, a growth arrest-specific 6 receptor agonist, targets the PGAM5–VDAC1 axis to maintain mitochondrial homeostasis, restore mitochondrial energy metabolism and suppress LPS-induced nectroposis in cardiomyocytes, thereby reversing cardiac injury^135^. These data highlight puerarin as a prospective therapeutic agent that targets specific mitophagy pathways. Olaparib, an anticancer drug, exerts potential protective effects against septic cardiac dysfunction by accelerating mitophagy flux and inhibiting ferroptosis^150^. A recent study showed that Po-Ge-Jiu-Xin decoction triggers PINK1/Parkin-mediated mitophagy, resulting in suppression of the inflammatory responses and myocardial apoptosis, indicating potential therapeutic benefits in sepsis-induced cardiomyopathy^151^. In addition, natural product Liang-Ge-San suppresses virus-induced inflammation in macrophages by upregulating α7 nicotinic acetylcholine receptor, which inhibits mitophagy in alveolar epithelial cells (MLE-12) and alleviates ALI and inflammation in mouse models infected with SARS-CoV-2 or H1N1, or treated with polyinosinic:polycytidylic acid^152^. Protopine, an alkaloid with anti-inflammatory and antioxidant properties, can ameliorate septic lung damage by downregulating mitophagy and the expression of PINK1/Parkin^153^. A list of antisepsis agents modulating mitophagy is summarized in Table 5.

**Table 5:** Agents to treat sepsis using mitophagy processes.

Agent	Infection model	Disease	Target	Biological responses	Outcomes	Ref.
Emodin	LPS-induced sepsis	Sepsis	Macrophage	Activates FUNDC1-mediated mitophagy	Protective effect against sepsis via inhibiting NLRP3 inflammasome activation	^146^
High concentration of H_2_ inhalation	CLP	Sepsis-associated encephalopathy	Hippocampal tissue	Activates PINK1/Parkin-mediated mitophagy and inhibits cGAS–STING–IRF3 pathway	Attenuates neuroinflammation	^147^
Bergapten	*E. coli* bacteremia	Sepsis with bacteremia	Macrophage	Activates mitophagy	Ameliorates tissue inflammation via inhibiting NLRP3 inflammasome activation	^148^
Dendrobine	CLP	Sepsis-associated AKI	Renal tubular epithelial cell	Enhances PINK1/Parkin-mediated mitophagy by upregulation of mitophagy-related proteins (PINK1, PARKIN, LC3B/A)	Kidney protective effect against sepsis	^149^
Puerarin	LPS-induced sepsis	Septic cardiomyopathy	Microvascular endothelial cell	Restores mitophagy activity via targeting the PGAM5–VDAC1 axis	Improves sepsis-induced coronary microvascular injury	^135^
Olaparib	CLP	Septic cardiomyopathy	Myocardial cell	Activates mitophagy	Reduces ferroptosis via improving mitochondrial quality	^150^
Po-Ge-Jiu-Xin decoction	CLP	Septic cardiomyopathy	Myocardial cell	Activates mitophagy	Ameliorates myocardium injury by preserving mitochondrial quality	^151^
Liang-Ge-San	SARS-CoV-2	Sepsis-associated ALI	Lung epithelial cell	Inhibits TNF-α-induced mitophagy	Inhibits TNF-α-induced mitochondrial damage and tissue injury	^152^
Protopine	CLP	Sepsis-associated ALI	Lung tissue	Inhibits mitophagy via downregulation of PINK1 expression	Prevents excessive mitophagy, cell death and ALI	^153^

*CLP* cecal ligation and puncture, *IRF3* interferon regulatory factor 3, *TNF-α* tumor necrosis factor alpha.

Thus, several reagents targeting mitophagy may serve as candidate drugs for treating sepsis or related pathologies, including ALI or acute respiratory distress syndrome, which are usually accompanied by viral or bacterial infections.

## Conclusion

Most pathogenic infections result in mitochondrial damage, energy deprivation and alterations in mitochondrial function and morphology, all of which trigger mitophagy. In addition to their effects on xenophagy modulation, various pathogens, including intracellular bacteria and viruses, have evolved sophisticated strategies to manipulate the host mitophagy machinery to support their persistence and survival. Increasing evidence has highlighted the pivotal role of mitophagy in regulating immune and inflammatory responses across diverse infectious conditions, including bacterial and viral infections and sepsis. Evidently, mitophagy plays dual roles in infection: it supports cellular and tissue homeostasis by removing damaged mitochondria and preventing excessive immune activation, while also creating an intracellular environment conducive to pathogen survival and replication, thereby facilitating parasitism. In this Review, we highlight evidence supporting both the protective and pathological roles of mitophagy in infection and sepsis. We also discuss recent progress in the development of mitophagy-targeting therapeutics aimed at either enhancing host defenses or inhibiting the pathogenic manipulation of mitophagy.

Despite these advances, many critical questions remain unanswered in this emerging field of host–pathogen interactions. For example, what types or modes of mitophagy are specifically required for pathogen survival or modulation of host inflammatory and cell death pathways? How do these processes vary depending on pathogen type and infection outcome? From the pathogen perspective, which specific microbial effectors interact with or hijack host mitophagy pathways to promote infection or evade immune detection? A broader understanding of how different pathogens—including viruses, bacteria, and parasites—exploit or disrupt mitophagy to evade immune surveillance is urgently required. Over the past decade, substantial progress has been made in elucidating the complex interplay between mitophagy and infectious agents, thereby increasing the therapeutic potential of mitophagy modulators in infections and sepsis. However, we are still in the early stages of developing selective mitophagy-inducing or inhibiting agents that can function as adjunctive therapeutics.

Therefore, modulating mitophagy represents a promising and underexplored strategy for combating intractable and emerging infectious diseases, as well as for controlling sepsis and its related complications. Future studies must clarify the molecular mechanisms by which mitophagy shapes innate and adaptive immune responses, and pathogens co-opt or disrupt these pathways to evade host defenses. Unraveling these mechanisms is essential for developing novel vaccines, therapeutics and immunomodulatory strategies that target mitophagy in infectious and inflammation-related diseases.
